# Vitamin D Deficiency and the Lung: Disease Initiator or Disease Modifier?

**DOI:** 10.3390/nu5082880

**Published:** 2013-07-26

**Authors:** Rachel E. Foong, Graeme R. Zosky

**Affiliations:** Centre for Child Health Research, Telethon Institute for Child Health Research, The University of Western Australia, 100 Roberts Road, Subiaco, Western Australia 6008, Australia; E-Mail: rfoong@ichr.uwa.edu.au

**Keywords:** vitamin D, asthma, COPD, lung cancer

## Abstract

Vitamin D deficiency is a global public health problem and has been associated with an increased incidence and severity of many diseases including diseases of the respiratory system. These associations have largely been demonstrated epidemiologically and have formed the basis of the justification for a large number of clinical supplementation trials with a view to improving disease outcomes. However, the trials that have been completed to date and the ongoing experimental studies that have attempted to demonstrate a mechanistic link between vitamin D deficiency and lung disease have been disappointing. This observation raises many questions regarding whether vitamin D deficiency is truly associated with disease pathogenesis, is only important in the exacerbation of disease or is simply an indirect biomarker of other disease mechanisms? In this review, we will briefly summarize our current understanding of the role of vitamin D in these processes with a focus on lung disease.

## 1. Introduction

The prevalence of vitamin D deficiency around the world is increasing. While the importance of vitamin D in bone health has been recognized for nearly two centuries [1], there has been growing recognition of the critical extra-skeletal roles for vitamin D in recent years [2]; with a particular focus on chronic disease. As such, there has been an explosion of interest in vitamin D across health disciplines including lung disease.

While vitamin D can be obtained from dietary sources (D_2_ isoform) the biggest contribution to an individual’s circulating levels of vitamin D, in most countries, is through endogenous production in the skin following exposure to ultraviolet(UV)-B radiation from the sun [2]. Exposure to UV-B (wavelength ~290–315 nm) converts 7-dehyrdrocholestrol in the skin into pre-vitamin D (D_3_ isoform) which spontaneously isomerizes into vitamin D [1]. This vitamin D, along with any vitamin D obtained through the diet, is converted into 25(OH)D in the liver [1]. Circulating levels of 25(OH)D are used as a marker of an individual’s vitamin D status [2] and, while there is some debate regarding what constitutes levels that are sufficient for normal physiological function, the current Institute of Medicine (IOM) guidelines from 2011 recommend 20 ng mL^−1^ (~50 nmol L^−1^) [3] which is based entirely on the levels required to maintain adequate bone health.

The serum levels of 25(OH)D are measured as a marker of vitamin D status due to its long half-life (~15 days) [4], however it should be acknowledged that 25(OH)D represents the reservoir available for production of the active form 1,25(OH)_2_D which is produced, primarily in the kidney, through enzymatic conversion by 25-hydroxyvitamin D-1α-hydroxylase [2]. As a result, there are several pathways that can influence an individual’s ability to produce and respond to adequate local quantities of the active form of vitamin D which is the true determinant of physiological function. 1,25(OH)_2_D interacts with vitamin D binding protein (VDBP) which facilitates transport of the molecule. 1,25(OH)_2_D enters the cell, binds to the nuclear vitamin D receptor and forms a complex with retinoid X receptor (RXR) [1]. This receptor complex then directs transcription of genes with a vitamin D response element (VDRE) [5].

It is important to recognise from the outset that, in reviewing the link between vitamin D and chronic lung disease, there are several challenges. The first of which is the widespread expression of the vitamin D receptor (VDR) across different cell types throughout the body and the high number of genes that contain a vitamin D response element (VDRE). This means that while there have been several studies showing epidemiological associations vitamin D and lung disease outcomes, we still have a poor understanding of the potential mechanisms involved due to the complex nature of the pathways involved. Likewise, the observation that most of our vitamin D is produced by exposure to UV radiation is critically important for the simple reason that UV-B is known to be an important immunomodulator in itself and can exert its effects through several non-vitamin D dependent pathways (reviewed in [6]). This means that, in epidemiological associations between vitamin D and chronic lung disease, it is possible that serum vitamin D levels are simply a surrogate marker for UV-B exposure and vitamin D is not on the causal pathway for disease outcomes.

In the context of chronic lung disease the potential association with vitamin D is further confounded by the strong association between physical activity, which is directly linked to an individual’s level of sun exposure [7] and disease severity [8,9], which means that it is unclear if vitamin D is simply an indirect marker of physical activity and, consequently, is an innocent bystander in disease pathogenesis. Thus, in many instances, it is uncertain whether vitamin D is critical in disease onset or is just an indirect biomarker for morbidity. One further issue that has not been addressed anywhere in the literature is the direct effect of inflammation on circulating 25(OH)D. Given that there is local upregulation of the conversion of 25(OH)D to 1,25(OH)_2_D during infection one might predict that immediate circulating store of 25(OH)D would be depleted. Thus, one intriguing possibility is that in the setting of chronic inflammation circulating vitamin D levels may be reduced as a direct result of the inflammatory response itself, which further confounds the positive association between vitamin D deficiency and disease severity.

In this review, we will briefly summarize our current knowledge regarding the link between vitamin D and several common chronic respiratory conditions in an attempt to examine the importance of vitamin D deficiency in disease onset, through disease progression (pathogenesis) and during the acute exacerbation of disease. In this review, we have chosen to focus on asthma, chronic obstructive pulmonary disease (COPD) and lung cancer as they collectively represent a significant public health burden. We will also briefly mention respiratory infections but primarily in the context of their importance in the exacerbation of disease. We acknowledge that by focusing on these diseases we will not be addressing the literature linking fibrotic disease (both cystic fibrosis and idiopathic pulmonary fibrosis) and vitamin deficiency. For interested readers these associations are reviewed elsewhere [10].

## 2. Respiratory Infections

Respiratory infections are a significant cause of morbidity and mortality worldwide. While respiratory infections *per se* are not typically considered in the category of “chronic” lung disease they can be important modifiers of disease progression and are key drivers of the exacerbation of many chronic lung diseases. As such, the importance of vitamin D in determining responses to respiratory infections will be briefly mentioned here (for more comprehensive reviews see [11,12,13]).

### Vitamin D and Respiratory Infections

It has long been recognised that one of the key non-skeletal effects of vitamin D is to modulate the immune response to pathogens. As part of the innate immune response, 1,25(OH)_2_D induces the production of antimicrobial peptides including cathelicidin and β-defensin 2. The promoter region of the genes coding for these two peptides contain vitamin D response elements, indicating 1,25(OH)_2_D-dependent regulation [14]. Cathelicidins and defensins have a broad spectrum of antimicrobial activity and kill bacteria by disruption of microbial membranes. In addition, they also act as chemoattractants for other inflammatory cells and contribute to wound repair [15]. An important study of *Mycobacterium tuberculosis* infection in human monocytes found that activation of toll-like receptor (TLR)1 and TLR2 [16], receptors responsible for recognizing microbial ligands, led to upregulation of the vitamin D receptor (VDR) and CYP27B1, the enzyme responsible for converting 25(OH)D to 1,25(OH)_2_D. The endogenous production of 1,25(OH)_2_D by CYP27B1 and subsequent action through the VDR led to the induction of cathelicidin, thus demonstrating a mechanism of the antimicrobial activity of vitamin D [16]. In addition to its immediate impact on the production of antimicrobial peptides vitamin D has been implicated in modifying the signaling pathways that bind respiratory viruses. For example, 1,25-dihydroxyvitamin D decreases the expression of ICAM-1, the major cellular receptor for human rhinovirus [17] in human umbilical vein endothelial cell cultures [18] and peripheral blood mononuclear cells [19]. Given the importance of rhinovirus in the exacerbation of both asthma and COPD this has important implications for lung health.

On the basis of these observations, along with a plethora of other studies that were not mentioned, it is clear that vitamin D is important in the immediate response to respiratory infections in experimental systems. This is supported epidemiologically by seasonal patterns in the prevalence of respiratory infections [20]. There have been several clinical trials, of varying caliber, that have assessed vitamin D supplementation which also point to a beneficial effect of vitamin D on responses to respiratory infection. It should be noted that these epidemiological associations remain controversial as they can be difficult to separate from other seasonal confounders, such as increased proximity to infected individuals during winter, and on balance the responses in clinical trials has been variable [21]. However, to date the weight of evidence points to an important role for vitamin D in modulating the response to respiratory infection both in terms of disease susceptibility and severity. Importantly respiratory infections have been implicated in the onset, progression and exacerbation of chronic lung diseases.

## 3. Asthma

Asthma is a chronic disorder of the conducting airways characterized by airway inflammation, airway remodeling and airway hyperresponsiveness (AHR) [22]. Asthma is a heterogeneous disease which, in the most common form, is associated with allergic sensitization [23]. There is emerging evidence that signs of disease, including airway remodeling, may be present early in life [24]. As such, if vitamin D is important in disease onset then it is most likely to exert its effect *in utero* or early in post-natal life. Much of the burden in asthma, both in terms of morbidity and mortality, is due to asthma exacerbations and the subset of asthmatics that do not respond to common therapies. Interestingly, there is evidence to support a role for vitamin D in both of these aspects of asthma although, again, the potential association between disease severity and physical activity needs to be considered. These issues will be discussed in more detail below.

### 3.1. Vitamin D and Asthma Onset

Recently it has been hypothesized that westernized patterns of behavior have caused the human population to spend more time indoors away from sun exposure, leading to an increase in asthma and allergy as a result of vitamin D deficiency [25]. The importance of sun exposure in asthma is supported by the positive correlation between latitude and asthma prevalence [26]. Since UV exposure decreases at distances further away from the equator these observations suggest that vitamin D may play a role in asthma pathogenesis. Data from an unselected community birth cohort study from Perth, Australia showed that low serum 25(OH)D levels at 6 years of age were predictive of subsequent atopy or asthma-associated phenotypes at 14 years of age in boys [27]. A cross-sectional Italian study of children with asthma found that 53.3% of the children surveyed were vitamin D-deficient with serum 25(OH)D levels less than 20 ng/mL. Lower vitamin D levels were associated with worse asthma control and lower lung function [28]. In another cross-sectional study from North America, 17% of children with asthma were vitamin D-deficient and there was a significant correlation between vitamin D levels and lung function as well as markers of atopy such as IgE levels and positive skin prick test responses [29]. In adults with asthma, low serum 25(OH)D levels are associated with lower lung function, increased AHR and reduced sensitivity to glucocorticoids [30]. Much of the data from these observational studies support the hypothesis that higher vitamin D levels lead to better asthma outcomes. However, as mentioned previously, vitamin D deficiency is often an indirect marker of other confounding factors such as physical activity, making it hard to determine a causal association between vitamin D status and asthma.

Birth cohort studies investigating the associations between maternal vitamin D status and asthma outcomes in children have shown that lower maternal dietary intake of vitamin D during pregnancy is associated with an increased risk of wheeze [31,32] and the development of asthma in children [33]. In contrast a birth cohort study from Finland also found that vitamin D supplementation in the first year of life was associated with an increased prevalence of asthma and atopy at 31 years of age [34]. However, vitamin D status in these studies was assessed using food questionnaires rather than directly measuring serum 25(OH)D concentrations. Gale *et al.* [35] reported that children whose mothers had serum 25(OH)D concentrations above 75 nmol/L had an increased risk of asthma at nine years of age (OR = 5.4 95% CI 1.09, 26.65; *p* = 0.038) [35]. However another recent study measuring serum 25(OH)D during late pregnancy did not find an association between maternal vitamin D status and risk of childhood asthma, wheeze or atopy at six years of age [36]. A Spanish birth cohort study also found no association between maternal vitamin D status during pregnancy and the incidence of wheeze or asthma but reported an inverse association with the risk of respiratory infection [37].

In another study low cord blood 25(OH)D levels were associated with an increased risk of respiratory infections at three months of age and wheeze by five years of age, but again were not associated with asthma incidence [38]. As such, the evidence to suggest that higher 25(OH)D levels reduce the incidence of asthma is conflicting and there is yet to be a study that convincingly demonstrates that vitamin D deficiency is implicated in the onset of asthma. However, the association between low levels of vitamin D, wheeze and respiratory infections appears to be more consistent. Given the strong positive association between the frequency and severity of early life respiratory infections and the risk for developing asthma [39] any increase in the respiratory infections as a result of primary vitamin D deficiency in early life is likely to impact on the likelihood of developing asthma. While this is an attractive hypothesis there has yet to be a study that has attempted to disentangle this complex pathway such that the current evidence implying a direct role for vitamin D in the onset of asthma is equivocal.

### 3.2. Vitamin D and Asthma Pathogenesis

Gupta and colleagues [40] measured serum 25(OH)D levels in a study which included children with moderate and steroid resistant asthma (STRA), as well as non-asthmatic subjects, and found that serum 25(OH)D levels were lowest in children with STRA. Consistent with other studies, the authors reported reduced lung function, increased corticosteroid use and asthma exacerbations with lower vitamin D levels in asthmatic children. Importantly, this study also found that low vitamin D levels were associated with an increase in ASM mass in children with STRA [40]. This study was the first study to demonstrate an association between serum vitamin D levels, lung function and structural changes *in vivo*, and the authors speculated that low vitamin D levels may be partly responsible for the increased ASM and reduced lung function in severe asthma. Importantly, *in vitro* studies support a role for vitamin D in airway remodeling. Increased proliferation of ASM cells exposed to serum from asthmatic patients is inhibited by 1,25(OH)_2_D [41]. 1,25(OH)_2_D also downregulates the expression of MMP-9 and a disintegrin and metalloprotease (ADAM33), previously identified as an asthma-susceptibility gene which is involved in airway remodeling. This is further supported by a study by Damera *et al.* demonstrating that 1,25(OH)_2_D can inhibit ASM cell proliferation in both normal and asthmatic subjects by preventing cell cycle progression [42]. *In vivo* and *in vitro* animal studies support an important role for vitamin D in modulating normal lung development [43,44] such that vitamin D deficiency impairs lung growth [45]. The important role for vitamin D in lung growth and development is often overlooked in favor of an immunomodulatory explanation for the apparent association between vitamin D deficiency and chronic lung disease. This is surprising given the strength of the effect of vitamin D on lung growth *in vivo* [45] and the role that structural deficits are likely to play in the onset of chronic lung disease and the prognosis of an individual who is genetically susceptible to the development of obstructive lung disease.

However, the immunomodulatory effects of vitamin D are also likely to be important in asthma pathogenesis and should not be ignored; ultimately an understanding of the impact of vitamin D deficiency on *both* immune function and normal growth and development is likely to result in the biggest advances in our understanding of the importance of this nutrient in chronic lung disease. The role of vitamin D on T cell responses has been well studied. T-cells, particularly T-helper (Th) 2 cells can contribute to the pathogenesis of asthma through the production of cytokines, such as IL-4, IL-5, IL-9 and IL-13. Secretion of these cytokines are essential for the class switching of B-cells to immunoglobin (Ig) E synthesis, the recruitment of mast cells and the maturation of eosinophils [46]. It is well established that 1,25(OH)2D inhibits Th1 cytokine production [47,48]. However, the effect of vitamin D on Th2 responses remains unclear. While 1,25(OH)2D has been shown to promote Th2 responses in murine T cells [47], Pichler *et al.* found that 1,25(OH)2D can also inhibit both Th1 and Th2 cytokine production from human cord blood T cells [49]. Studies investigating the effects of 1,25(OH)_2_D supplementation has also produced conflicting results. One study found that 1,25(OH)_2_D can inhibit the inflammatory response by treatment with 1,25(OH)_2_D at the onset of exposure to the experimental allergen ovalbumin (OVA) in mice [50]. Another study by Matheu *et al.* found that priming with 1,25(OH)_2_D prior to OVA sensitization resulted in enhanced antigen-specific IL-4, IL-13 and IgE production, but inhibited IL-5 release and eosinophilia [51]. The authors went on to investigate whether these effects were dependent on the timing of vitamin D treatment, and found that eosinophil recruitment was inhibited when 1,25(OH)_2_D was administered at a later stage in the exposure protocol. This work is supported by Gorman and colleagues [52] who showed that vitamin D deficiency in a mouse model of OVA exposure suppresses allergic responses in a sex dependent manner. Interestingly, the apparent sensitivity of male mice to the effects of vitamin D deficiency was associated with an increase in bacteria levels in the lung implying a role for vitamin D induced modulation of the microbiome in regulating asthma like responses in the airway. This is an area that warrants further investigation.

There is certainly mounting evidence for a role of vitamin D in altering the pathogenesis of asthma through modulation of T cell driven immune responses, however there is still clearly work to do in this field.

### 3.3. Vitamin D and Asthma Exacerbations

Severe asthma exacerbations may require hospitalization and account for much of the burden of asthma [53]. In a cross-sectional study of asthmatic children in Costa Rica between the ages of 6 and 14 years, higher levels of vitamin D were associated with reduced asthma exacerbations as determined by reduced odds of hospitalizations or emergency department visits (OR = 0.05 95% CI 0.004, 0.71; *p* = 0.03), as well as lower serum IgE, eosinophil counts and inhaled steroid use [54]. Brehm *et al.* [55] followed up these results with a prospective study of serum vitamin D levels and the subsequent development of severe asthma exacerbations over a 4-year period in North American asthmatic children. This study confirmed the findings from the Costa Rican study showing that vitamin D levels less than 30 ng/mL were associated with higher odds of asthma exacerbations (OR = 1.5 95% CI 1.1, 1.9; *p* = 0.01). Furthermore, children who were vitamin D insufficient, regardless of whether they did or did not receive inhaled steroids, had an increased risk of exacerbation compared with children who received inhaled steroids and had sufficient levels of vitamin D, indicating a role for vitamin D in enhancing steroid responsiveness [55]. Another study conducted in Puerto Rican children with and without asthma reported higher odds of asthma exacerbations (OR = 2.6 95% CI 1.5, 4.9; *p* = 0.001) and atopy, and lower FEV_1_/FVC in children with asthma who were vitamin D insufficient. There was no association between lung function and atopy and vitamin D insufficiency in children without asthma suggesting that vitamin D influences asthma exacerbations via mechanisms unrelated to *allergic* immune responses or lung structure [56].

One of the mechanisms in which vitamin D deficiency may contribute to asthma exacerbations is by reducing steroid responsiveness. Searing and colleagues [29] reported an inverse correlation between corticosteroid use and vitamin D levels in asthmatic children. Furthermore, vitamin D was able to restore corticosteroid action in an experimental model of steroid resistance [29]. Inhaled corticosteroids have a protective effect on severe asthma exacerbations and inhibit the synthesis of Th2 cytokines, which are implicated in asthma pathogenesis, and induce IL-10, a potent anti-inflammatory cytokine in airway epithelial cells. Since severe asthmatics are less responsive to corticosteroids compared with mild asthmatics, corticosteroid insensitivity may be a mechanism contributing to asthma severity [57]. Regulatory T cells from patients with severe therapy-resistant asthma do not have an increase in IL-10 following corticosteroid exposure. However, the administration of vitamin D may overcome this deficit in IL-10 production [58]. Microarray studies have also revealed that stimulation of ASM cells with the VDR ligand, 1,25(OH)_2_D, upregulates the expression of two genes coding for glucose-6-phosphate dehydrogenase and 1β-hydroxysteroid dehydrogenase type 1 enzyme, both of which are responsible for corticosteroid activation [59]. These data suggest that vitamin D supplementation could be used as an adjunct therapy to overcome steroid resistance in severe asthma.

While there is certainly emerging evidence of a role for vitamin D deficiency in steroid resistance, which may impact on the *propensity* for acute exacerbations, the important role of vitamin D in responses to infection also deserves recognition. This is particularly important given that almost all acute exacerbations of asthma that require hospitalization in children [60] are associated with an acute viral infection. As highlighted earlier there are a plethora of studies on the impact of vitamin D on responses to infection, however there are very few (if any) studies that have systematically assessed the causal role of vitamin D deficiency in modulating the *severity* and/or *duration* of response to viral infection during an acute asthma exacerbation. Of course, it is possible that this explains the observations from the studies described above showing an association between vitamin D and acute exacerbation outcomes, however whether this is due to an altered response to viral infection is yet to be elucidated.

## 4. Chronic Obstructive Pulmonary Disease (COPD)

COPD is a chronic inflammatory airway disease characterized by progressive destruction of the lung parenchyma (emphysema) and/or chronic inflammation of the small airways leading to hyperinflation and fixed airflow obstruction [61,62]. The pattern of disease onset, progression and the mechanisms leading to exacerbation in COPD can be difficult to dissect due to the multitude of factors that contribute to pathology in an individual patient. While COPD is primarily associated with smoking and, as such, has been treated as an “adult” disease, there is increasing recognition of the importance of early life factors in influencing the risk of developing disease [63,64]; particularly early life exposures that might influence immune function and/or lung growth. Thus, like asthma, vitamin D has the potential to influence disease onset by impacting on the lung early in life. The potential association between COPD and vitamin D is perhaps even more complex than that described for asthma due to the significance of muscular and bone related co-morbidities, which are highly influenced by vitamin D. As such, epidemiological associations between vitamin D and COPD outcomes are highly influenced by confounders.

### 4.1. Vitamin D, COPD and Musculoskeletal Co-Morbidities

The high prevalence of osteoporosis and osteopenia in COPD patients [65] may be an indication of a link between vitamin D deficiency and COPD. Osteoporosis and osteopenia are characterized by low bone mineral density partly due to reduced calcium intake and absorption. Vitamin D, together with parathyroid hormone increases intestinal calcium absorption to maintain normal calcium levels [66]. Janssens *et al.* [67] reported a high prevalence of vitamin D deficiency in patients with COPD from a cohort which included age, sex and smoking-matched controls. Serum 25(OH)D levels correlated with lung function as measured by FEV_1_ in COPD patients, but not healthy smokers [67]. A previous study also found that low vitamin D levels were common in a small cohort of patients with COPD awaiting lung transplantation [68]. Similar associations between serum 25(OH)D with FEV_1_ and FVC were reported in a large cross-sectional study from the third National Health and Nutrition Examination Survey (NHANES) [69]. Although there was no correlation with COPD, the association between serum 25(OH)D and FEV_1_ was increased in smokers and ex-smokers compared with non-smokers. Another recent study demonstrated a positive association between dietary vitamin D intake and FEV_1_, FEV_1_/FVC and an inverse association with COPD incidence [70]. However, as already suggested, these associations must be treated with additional caution due to the combined influence of the link between disease severity, physical activity levels and vitamin D and the strong link between musculoskeletal abnormalities and COPD.

### 4.2. Vitamin D and COPD Onset

When attempting to describe the impact of vitamin D on the onset of COPD there are two paradigms to consider; the early life origins of disease and the primary response to an individual’s first exposure/s to cigarette smoke. In both cases, data on the impact of vitamin D on these pathways for disease initiation is limited.

Like asthma, any factors that result in an early life deficit in lung function are likely to impact on disease morbidity (and mortality) later life. This is because lung function tends to follow a trajectory [71,72] whereby any deficit, relative to the population, is maintained throughout life such that the threshold for limitations in lung function following a respiratory insult is lowered. While there have been no epidemiological studies showing a link between early life vitamin D deficiency and COPD (or asthma), *in vivo* and *in vitro* experimental data suggests an important role for vitamin D in lung development. For example, vitamin D has been shown to increase the synthesis of surfactant in alveolar type II cells [43] and modulate epithelial-mesenchymal interactions [44] in the developing rat lung. More recently, it has been shown *in vivo* that vitamin D deficiency *in utero* and in early life, in the absence of hypocalcaemia, alters lung development in mice resulting in deficits in lung volume and impaired lung mechanics [45]. Together these observations implicate vitamin D for a role in normal lung growth. As a result, vitamin D deficiency has the potential to impact on the development of chronic lung disease in early although direct evidence for this in human studies is currently lacking.

Likewise, there have been almost no studies that have examined the impact of vitamin D on the primary response to cigarette smoke. One study has shown that exposing cells in culture to cigarette smoke inhibits vitamin D induced translocation of the nuclear VDR [73], however this appears to be the only evidence that cigarette smoke has a direct impact on vitamin D pathways. While acknowledging that work in this area is limited the potential upregulation of VDR in response to an insult has important implications when considering the validity of measuring 25(OH)D alone when examining disease associations. There is clearly more work that needs to be done on the impact of vitamin D on the onset of COPD.

### 4.3. Vitamin D and COPD Pathogenesis

COPD is generally seen as a disease affecting older individuals as a result of cigarette smoking. However about 20% of COPD cases occur in non-smokers and not all smokers develop COPD, indicating other contributors to disease pathogenesis [62]. A study investigating the risk factors associated with early inception of COPD measured lung function in a European cohort of young ages at ages 20 to 44 years, and again 8 to 9 years later. This study found that cigarette smoking remained the main cause of COPD in these young adults, however the same factors associated with asthma risk such as AHR, a family history of asthma and childhood respiratory infections were also risk factors for COPD [74]. As discussed earlier, these data are consistent with a growing body of evidence for early life origins of many chronic lung diseases of adulthood. Like asthma, there is an increase in ASM in the airways of COPD patients, predominantly in the small airways [75]. Thus, one tantalizing hypothesis for the common association between vitamin D deficiency and an increased incidence of asthma and COPD is through the impact of vitamin D on primary lung structure.

In COPD, vitamin D deficiency is often thought to be a consequence of the disease rather than the cause since COPD patients have a reduced capacity for vitamin D synthesis due to their aging skin and are more likely to spend less time outdoors. The data from the NHANES III study however suggests that vitamin D status directly affects lung function *per se* [69]. Genetic studies have also found a link between COPD and variants of the vitamin D binding protein (VDBP). VDBP is the major carrier protein for vitamin D and binds circulating 25(OH)D and 1,25(OH)2D with high affinity. However it is now known to have other functions including macrophage activation and can augment monocyte and neutrophil chemotaxis [5], both of which play an important role in COPD pathogenesis. Single nucleotide polymorphisms (SNPs) of the VDBP gene (*GC*), namely rs7041 and rs4588, produce the Gc1 and Gc2 variants which have different binding affinities for 25(OH)D. Janssens *et al.* [67] reported that the rs7041 variant Gc1S predicted 25(OH)D levels in COPD patients and was a genetic risk factor for the disease. Another study of vitamin D levels and alveolar macrophage function reported lower lung function and increased macrophage activation with higher levels of VDBP in the airways [76]. The Gc2 variant was also protective against COPD and a potential explanation for this is that Gc2 is less able to activate macrophages. Macrophage accumulation and activation in the COPD lung leads to the release of neutrophil chemoattractants, which may contribute to lung damage. Together the data suggests that genetic variants of VDBP may protect against COPD pathogenesis.

MMPs also have a potential role in the progression of COPD. MMP-9 is increased in the sputum of COPD patients [77] and MMP-9 activity is known to enhance the degradation of the lung parenchyma thus contributing to the emphysema phenotype observed in COPD. A study using VDR knockout mice reported emphysema and reduced lung function in these mice together with increased neutrophil and macrophage influx in the lung as well as upregulation of MMP-2, MMP-9 and MMP-12, suggesting that the lack of VDR activates pathways that are associated with COPD pathogenesis [78].

### 4.4. Vitamin D and COPD Exacerbations

COPD exacerbations are important events in the natural history of the disease. A recent consensus statement defined COPD exacerbations as “*a sustained worsening of the patient’s condition, from the stable state and beyond normal day to day variations that is acute in onset and necessitates a change in regular medication in a patient with underlying COPD*” [62,79]. Exacerbations have been described as clinical manifestations of increased inflammation and a key finding is an increase in neutrophils in the sputum during acute exacerbations [80]. The majority of COPD exacerbations are caused by viral and/or bacterial infections of the tracheobronchial tree [81]. Air pollution induces oxidative stress and is another important cause of exacerbations, however there is also a large proportion of exacerbations with no identifiable cause [62].

An analysis of sputum samples from 56 COPD patients in East London experiencing an exacerbation found that about 70% of exacerbations were associated with a bacterial pathogen, while 20% was associated with rhinovirus infection. In addition, bacterial and viral infections interact to cause more severe exacerbations [82]. In an experimental study where patients with COPD were infected with a low dose of rhinovirus, clinical features of an acute exacerbation were induced [83]. Subjects with COPD had greater airflow obstruction and neutrophilic inflammation compared with controls, demonstrating an important role for rhinovirus infections in COPD exacerbations. In contrast, another study from London investigating whether higher vitamin D levels reduced exacerbation frequency as well as susceptibility to human rhinovirus did not find any associations [84].

A study of North American patients with severe COPD found that more than 40% had serum 25(OH)D levels less than 20 ng/mL, however there was no association between baseline 25(OH)D levels and time to first acute exacerbation [85]. Another Norwegian study consisting of subjects with and without COPD found a high risk for vitamin D deficiency in COPD patients however again there was no association with self-reported exacerbation frequency [86]. Recent data from a randomized control trial of supplementation with high doses of vitamin D in a cohort of COPD patients did not reduce the incidence of exacerbations [87]. Thus, despite the evidence that vitamin D can reduce *infections* that may associated with acute exacerbations, current data does not support a role for vitamin D in preventing COPD exacerbations.

## 5. Lung Cancer

Lung cancer is the leading cause of cancer mortality in both men and women worldwide [88]. Lung cancer can be divided into two major classes, non-small-cell lung cancer (NSCLC), which accounts for 85% of all lung cancer and small-cell lung cancer (SCLC), representing 15% of cases. NSCLC can be further divided into adenocarcinoma, squamous cell carcinoma and large cell lung carcinoma [89]. While the main cause of lung cancer is cigarette smoking, an estimated 25% of cases also occur in non-smokers, most often in the form of adenocarcinomas [90]. Vitamin D has been studied extensively in many cancer settings and there is strong evidence to suggest that vitamin D is anti-tumorigenic [91].

### 5.1. Vitamin D and Lung Cancer Onset

The anti-tumorigenic activities of vitamin D are thought to be initiated via the binding of 1,25(OH)_2_D to the VDR. These mechanisms include inhibition of lung cancer cell proliferation, promoting apoptosis and reducing angiogenesis [92,93]. A recent *in vitro* study demonstrated that 1,25(OH)_2_D mediated G_0_/G_1_ cell cycle arrest via downregulation of cyclins which promote entry into the S phase of the cell cycle [94]. 1,25(OH)_2_D may prevent angiogenesis by reducing the secretion of vascular endothelial growth factor (VEGF), which is known to induce activation, migration and proliferation of endothelial cells [93]. MMP-2, MMP-9 and parathyroid hormone related protein (PTHrP) expression and production is also reduced in lung carcinoma cells treated with 1,25(OH)_2_D [93]. This may be an important mechanism since MMPs and PTHrP are also important factors for tumor invasion. The question is whether vitamin D only suppresses growth of established tumors or inhibits the development of lung cancer in the first place? A study in a mouse model of lung cancer found that 1,25(OH)_2_D supplementation decreased tumor incidence and significantly decreased tumor multiplicity in a dose-dependent manner [95]. 1,25(OH)_2_D can also inhibit metastatic growth of lung cancer cells *in vivo* [96], suggesting that maintaining adequate levels of vitamin D may prevent lung cancer pathogenesis. However, more clinical studies are required to determine if vitamin D can prevent carcinogenesis although the studies to date suggest that vitamin D status has an impact on the initiation of tumor growth.

### 5.2. Vitamin D and Lung Cancer Progression

Vitamin D deficiency is associated with an increased risk of developing colon, prostate and breast cancer, as well as a higher mortality due to these cancers [2]. A prospective cohort study found that serum 25(OH)D levels were inversely associated with lung cancer incidence in women (RR = 0.16 95% CI 0.04, 0.59; *p* < 0.001) and participants less than 50 years of age (RR = 0.34 95% CI 0.13, 0.90; *p* = 0.04), but not men (RR = 1.03 95% CI 0.59, 1.82; *p* = 0.81) and older participants (RR = 0.92 95% CI 0.50, 1.70; *p* = 0.79) [97]. Another prospective study of lung cancer risk in male smokers from Finland did not find an association between serum 25(OH)D levels and lung cancer risk, however a 10 nmol/L increase in serum 25(OH)D during the darker season was associated with a lower risk (OR = 0.89 95% CI 0.81, 0.98; *p* = 0.02) [98]. Given the high morbidity associated with cancer it is likely that in many instances the associations between lung cancer and vitamin D status may be due to a lack of sun exposure as a result of low physical activity. However, while epidemiological studies with lung cancer have not been entirely convincing, studies using animal models have found that vitamin D-deficient mice [99] and VDR knockout mice [100,101] have enhanced tumor growth in many tumor types including lung cancer [96]. 1,25(OH)_2_D has also been reported to have effects on cell proliferation and apoptosis and can inhibit tumor growth in colon, breast and prostate cells [102]. The enzyme responsible for breaking down 1,25(OH)_2_D, CYP24A1, is expressed in NSCLC cell lines, but not normal lung epithelial cells. Furthermore, several studies have found that expression of CYP24A1 is much higher in primary lung tumors compared with normal lung tissue samples, suggesting that the increased breakdown of 1,25(OH)_2_D may have inhibited its anti-proliferative effects [94,103,104]. Hansdottir *et al.* [105] also found reduced CYP27B1 expression in lung cancer derived cells compared with primary lung epithelial cells, indicating that the cancer cells did not convert 25(OH)D to 1,25(OH)_2_D. High CYP24A1 and low CYP27B1 both result in lower levels of 1,25(OH)_2_D, so, taken together, these studies suggest that low 1,25(OH)_2_D levels may be important in lung cancer progression.

### 5.3. Vitamin D and Lung Cancer Mortality

Data showing that vitamin D can inhibit lung cancer cell proliferation has prompted investigations into the link between vitamin D status and lung cancer mortality. Data from the NHANES III study did not show an association between vitamin D status and overall lung cancer mortality, but did demonstrate that serum 25(OH)D levels less than 44 nmol/L were associated with a decreased risk of mortality in non-smokers (HR = 0.53 95% CI 0.31, 0.92) [106]. Studies in early stage NSCLC patients found that increased UVB exposure, as determined by surgical resections during summer, and higher vitamin D intake [107], as well as high circulating 25(OH)D serum levels resulted in improved survival [108]. Another Turkish study also demonstrated similar findings of shorter survival in NSCLC patients who underwent resection in winter compared to those operated on in summer [109]. Despite not measuring the vitamin D status of patients, the authors report that a polymorphism in the VDR gene was an independent prognostic indicator in resected NSCLC patients. Heist *et al.* [110] also reported that having the *T* allele of the *VDR* > *Fok1* > *T* polymorphism was associated with worse survival, but vitamin D status had no effect on survival in patients who had advanced NSCLC. High nuclear VDR expression was also associated with improved overall survival in NSCLC patients (adjusted HR = 0.36 95% CI 0.17, 0.79; *p* = 0.011) [111], while overexpression of CYP24A1 resulted in poorer survival (HR = 2.1 95% CI 1.14, 3.75; p = 0.001) [104]. A recent study has also demonstrated that VDBP expression is low in lung cancer tissue and low circulating VDBP was a predictor of subsequent death from lung cancer in patients [112], indicating that VDBP is also an important independent factor in determining better survival outcomes in lung cancer patients.

## 6. Conclusions

The growing prevalence of vitamin D deficiency around the globe is a significant public health concern. Based on the weight of evidence to date it is clear that vitamin D is important in lung disease. In moving forward to address this problem it is important that we understand this association better in order to identify (1) the minimum (and maximum) vitamin D levels required for normal lung growth, development and immune function and (2) when to intervene if necessary. To that end, we need a thorough understanding of the importance of vitamin D in determining the onset of disease, progression of disease and, in the case of asthma and COPD, the exacerbation of disease. To date the bulk of the studies have focused on associations between an individual’s *current* vitamin D and disease status which is not sufficient to adequately inform public health policy in order to ameliorate the vitamin D deficiency induced burden of respiratory disease in the community.
